# Racism in German medical education – Perspectives of racialized physicians and medical students

**DOI:** 10.1093/eurpub/ckac131.106

**Published:** 2022-10-25

**Authors:** H Vogt

**Affiliations:** NaDiRa, DeZIM, Berlin, Germany

## Abstract

**Background:**

The impact of racism on health and the quality of health care services for racialized patients have been the object of prior research. The experiences of racialized medical students and physicians, who operate in a field of tension between natural sciences and practical application and between a ‘medical habitus’ and exclusionary discrimination, have hardly been examined to date. The education of (future) physicians is an especially fruitful research area in the context of the study of institutional racism in the health care system, as informal everyday experiences come together with formal knowledge and normative learning.

**Methods:**

Based on expert consultations and preliminary interviews with civic stakeholders, teaching and learning materials in German medical studies were randomly sampled and used as a starting point for qualitative guided interviews with physicians and medical students in Germany who are themselves affected by racism. The first steps of the thematic analysis of these interviews are reflected upon and further developed in focus group discussions with the interviewees.

**Results:**

The study is particularly concerned with the question of how certain dimensions of racism in the health care sector are related, and how racist normativity appears in this context. This is concretized in the relationship between formal and informal medical curricula as well as in the interweaving of everyday experiences and teaching materials.

**Conclusions:**

There are different dimensions of relation between a hegemonic normativity in the medical curriculum and the everyday experiences of racialized medical students and physicians in Germany. Politics, faculties, publishers, and civic society may be the target of several recommendations for action regarding those diverse dimensions, e.g. the line between omission and stereotyping of several patient groups.

**Key messages:**

• Hegemonic normativity in German medical education is an important and challenging issue. There are several institutional levels of medical knowledge reproducing racism in the health care sector.

• Both racialized physicians and racialized patients are affected by symbolic and material forms of racism in the German health care system, which are interweaving and crucial on diverse levels.

